# Novel cross-talk between IGF-IR and DDR1 regulates IGF-IR trafficking, signaling and biological responses

**DOI:** 10.18632/oncotarget.3177

**Published:** 2015-03-28

**Authors:** Roberta Malaguarnera, Maria Luisa Nicolosi, Antonella Sacco, Alaide Morcavallo, Veronica Vella, Concetta Voci, Michela Spatuzza, Shi-Qiong Xu, Renato V. Iozzo, Riccardo Vigneri, Andrea Morrione, Antonino Belfiore

**Affiliations:** ^1^ Endocrinology, Department of Health Sciences, University Magna Graecia of Catanzaro, Catanzaro, Italy; ^2^ Motor Sciences, School of Human and Social Sciences, Kore University of Enna, Enna, Italy; ^3^ Institute of Neurological Sciences, National Research Council, Catania, Italy; ^4^ Department of Urology and Biology of Prostate Cancer Program, Kimmel Cancer Center, Thomas Jefferson University, Philadelphia, PA, USA; ^5^ Department of Pathology, Anatomy and Cell Biology and Cancer Cell Biology and Signaling Program, Kimmel Cancer Center, Thomas Jefferson University, Philadelphia, PA, USA; ^6^ Endocrinology, Department of Clinical and Sperimental Medicine, University of Catania, Garibaldi-Nesima Hospital, Catania, Italy

**Keywords:** IGF-IR, insulin-like growth factor-I receptor, DDR1, breast cancer

## Abstract

The insulin-like growth factor-I receptor (IGF-IR), plays a key role in regulating mammalian development and growth, and is frequently deregulated in cancer contributing to tumor initiation and progression. Discoidin domain receptor 1 (DDR1), a collagen receptor tyrosine-kinase, is as well frequently overexpressed in cancer and implicated in cancer progression. Thus, we investigated whether a functional cross-talk between the IGF-IR and DDR1 exists and plays any role in cancer progression.

Using human breast cancer cells we found that DDR1 constitutively associated with the IGF-IR. However, this interaction was enhanced by IGF-I stimulation, which promoted rapid DDR1 tyrosine-phosphorylation and co-internalization with the IGF-IR. Significantly, DDR1 was critical for IGF-IR endocytosis and trafficking into early endosomes, IGF-IR protein expression and IGF-I intracellular signaling and biological effects, including cell proliferation, migration and colony formation. These biological responses were inhibited by DDR1 silencing and enhanced by DDR1 overexpression.

Experiments in mouse fibroblasts co-transfected with the human IGF-IR and DDR1 gave similar results and indicated that, in the absence of IGF-IR, collagen-dependent phosphorylation of DDR1 is impaired.

These results demonstrate a critical role of DDR1 in the regulation of IGF-IR action, and identify DDR1 as a novel important target for breast cancers that overexpress IGF-IR.

## INTRODUCTION

The type I IGF-I receptor (IGF-IR) binds with high affinity both insulin like growth factors I and II (IGF-I and IGF-II), and has a crucial role in the regulation of mammalian development and growth [1–3]. IGF-IR and its ligands are frequently dysregulated in cancer and affect not only the early phases of carcinogenesis but also cancer progression and cancer resistance to therapies [4–9]. IGF-II, and to a lesser extent IGF-I, bind also to the isoform A of the insulin receptor (IR-A), which is highly homolog to the IGF-IR [10, 11]. The IR-A, considered the fetal IR isoform, primarily mediates the mitogenic effects of IGF-II and insulin, and is implicated in development and cancer [12], while the second IR isoform (IR-B) is prevalently involved in glucose metabolism of insulin target organs [8].

We have previously demonstrated that IR-A associates with discoidin domain receptors (DDRs) after IGF-II stimulation [13]. DDRs are membrane receptor tyrosine-kinases (RTKs) that bind to and are activated by various forms of collagen, and include two family members, DDR1 and DDR2, which are encoded by different genes [14, 15]. DDRs are characterized by an extracellular discoidin domain and by a long juxtamembrane region. DDR1 is present in five isoforms (DDR1a–e) widely expressed in normal epithelium, while DDR2, which has no isoforms, is expressed in stromal and smooth muscle cells [15, 16]. DDRs have 13–15 tyrosine residues in their cytoplasmic domain, which serve as binding sites of Src-homology-2 (SH2) and phosphotyrosine binding (PTB) domain-containing molecules. DDR1 is the better characterized in this respect. It interacts at tyrosine 513 with the PTB domain of ShcA [17], but also with several other molecules, including the tyrosine phosphatase Shp-2, the adapter protein Nck2, and the regulatory subunit of phosphatidyl-inositol-3 kinase, p85 [18]. Unlike other TKRs, DDRs have slow activation kinetics, and tyrosine-phosphorylation usually requires hours after collagen binding [17]. High affinity binding to collagen requires DDR1 dimerization [19, 20], but a percentage of DDR1 molecules may dimerize in ligand-independent manner [21].

The biological role of DDRs has not been fully elucidated. However, DDRs have important roles in the regulation of cell to matrix adhesion, as well as cell proliferation and migration [22, 23]. An important role of DDR1 in growth regulation is suggested by data obtained in *DDR1*-null mice indicating that mutant animals were viable but smaller in size than control littermates. The majority of mutant females were unable to bear offspring due to a lack of proper blastocyst implantation into the uterine wall [24]. As far as DDR1 isoforms are concerned, the a and the b isoforms are the most expressed, and limited evidence indicates that these two isoforms might elicit somehow different biological responses [14, 16, 22, 25, 26]. However, data in this regard are scanty and not univocal. DDR1d and e-isoforms are truncated and kinase defective variants [27].

Noteworthy, DDRs definitely play a role in cancer. In fact, DDR1 overexpression has been reported in several malignancies, where it may have a role in epithelial-mesenchymal transition and cancer progression [25]. In spite of observations indicating that IGF-IR and DDRs are both important regulators of growth, cell adhesion and migration, a cross-talk between DDR1 and IGF-IR has not been previously established.

In the present study we show that DDR1 and IGF-IR do indeed functionally interact. This interaction is enhanced by IGF-I stimulation, which also promotes internalization of the IGF-IR/DDR1 complex. DDR1 regulates IGF-IR trafficking, and increases IGF-IR protein expression at post-translational level. Accordingly, ligand-activated IGF-IR downstream signaling and biological responses are impaired by DDR1 silencing and enhanced by DDR1 overexpression. Intriguingly, IGF-IR activation not only stimulates collagen-independent DDR1 phosphorylation, but also plays a role in collagen-induced DDR1 phosphorylation.

These data provide novel mechanistic insights onto DDR1 action as an important modulator of IGF-IR function in physiology and disease.

## RESULTS

### DDR1 and IGF-IR expression in cultured cells

In order to determine whether DDR1 may functionally interact with the IGF-IR, we first evaluated by immunoblot DDR1 and IGF-IR expression in a panel of IGF-I-responsive human breast cancer cell lines (MCF-7, T47D, ZR-75, MDA-MB-157, MDA-MB-231, BT-474), and in human HepG2 hepatoblastoma cells. We also evaluated R^−^ mouse fibroblasts, which lack expression of the *IGF-IR*, and R^−^-derived cell lines, either transfected with the human IGF-IR (R^+^ cells), with the human DDR1 (R^−^/DDR1 cells) or the corresponding empty vector (R^−^/EV) as controls.

**Figure 1:**
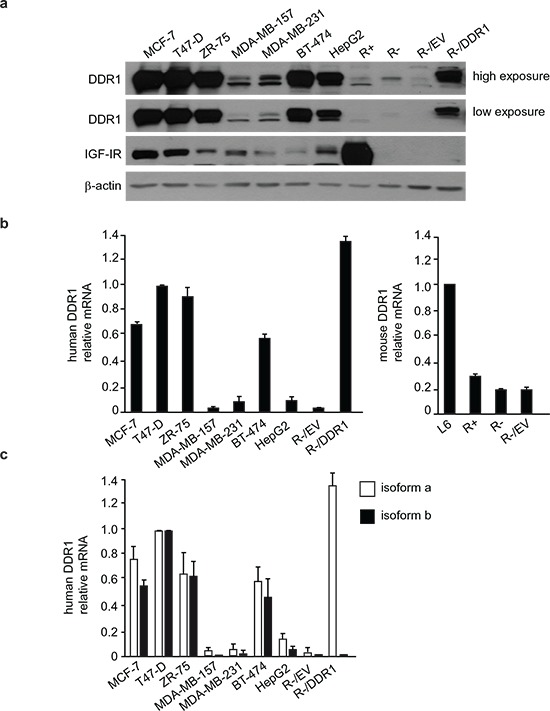
DDR1 and IGF-IR expression in a panel of cultured cells **(a)** *DDR1 and IGF-IR protein expression.* A panel of cell lines including human breast cancer (MCF-7, T47D, ZR-75, MDA-MB-157, MDA-MB-231 and BT-474), human hepatoblastoma (HepG2), and mouse embryo fibroblasts (R^−^, lacking endogenous IGF-IR, and R^+^, stably transfected with the human IGF-IR cDNA) were analyzed by western immunoblot for DDR1 and IGF-IR expression using polyclonal antibodies against the C-terminus of DDR1 and C-terminus of IGF-IR, as indicated. R^−^ cells stably transfected with either an empty vector (R^−^/EV) or with plasmid encoding human DDR1 isoform a (R^−^/DDR1), were used as controls. β-actin antibody was used as control for protein loading. A representative blot of three independent experiments is shown. **(b)** *qRT-PCR analysis of DDR1 mRNA*. Human and mouse DDR1 mRNA levels were evaluated in all cell lines shown in panel (a). L6 myoblasts were used as reference for DDR1 expression in mouse fibroblasts. Normalization was done using human β-actin or mouse GAPDH as housekeeping control genes. Data are presented as the mean ± SEM (error bars) from three independent experiments. **(c)** *Relative quantification of DDR1 isoform a and b mRNA*. Human DDR1 isoform a and b mRNA levels were evaluated in the same cells as in panel (b). Controls were used as in (b). Data are presented as the mean ± SEM of three independent experiments.

MCF-7, T47D, ZR-75, BT-474 and HepG2 cells showed high expression of DDR1 protein while MDA-MB-157 and MDA-MB-231 showed lower DDR1 expression (Figure 1a). R^−^ and R^−^-derived cell lines were characterized by very low DDR1 content (Figure 1a). Among cancer cells, MCF-7 and T47D showed the highest IGF-IR levels while the lowest levels were detected in MDA-MB-231 and BT-474. As expected, R^−^ cells and derivatives did not express the IGF-IR, with the exception of R^+^ cells, which expressed high IGF-IR levels [28] (Figure 1a).

Analysis of DDR1 mRNA expression by quantitative real-time RT-PCR was in agreement with the immunoblot results, with the exception of HepG2 that showed lower mRNA levels than expected (Figure 1b). To evaluate whether the main DDR1 isoforms, DDR1a and DDR1b, were both expressed, we used quantitative RT-PCR with isoform-specific primers, and determined that both these isoforms were expressed at similar levels in all cell lines (Figure 1c).

Because of their specific features, MCF-7, BT-474 and MDA-MB-231 breast cancer cells were chosen for subsequent experiments. All these cells have ductal characteristics and metastatic potential, and all respond to IGF-I. MCF-7 and BT-474 are estrogen receptor positive, while BT-474 cells are also HER-2 positive and tamoxifen resistant. Both cell lines have high DDR1 and IGF-IR levels. MDA-MB-231 have characteristics of triple negative cells and express lower levels of both DDR1 and IGF-IR.

### DDR1 associates with the IGF-IR constitutively, and the association is enhanced by IGF-I

**Figure 2:**
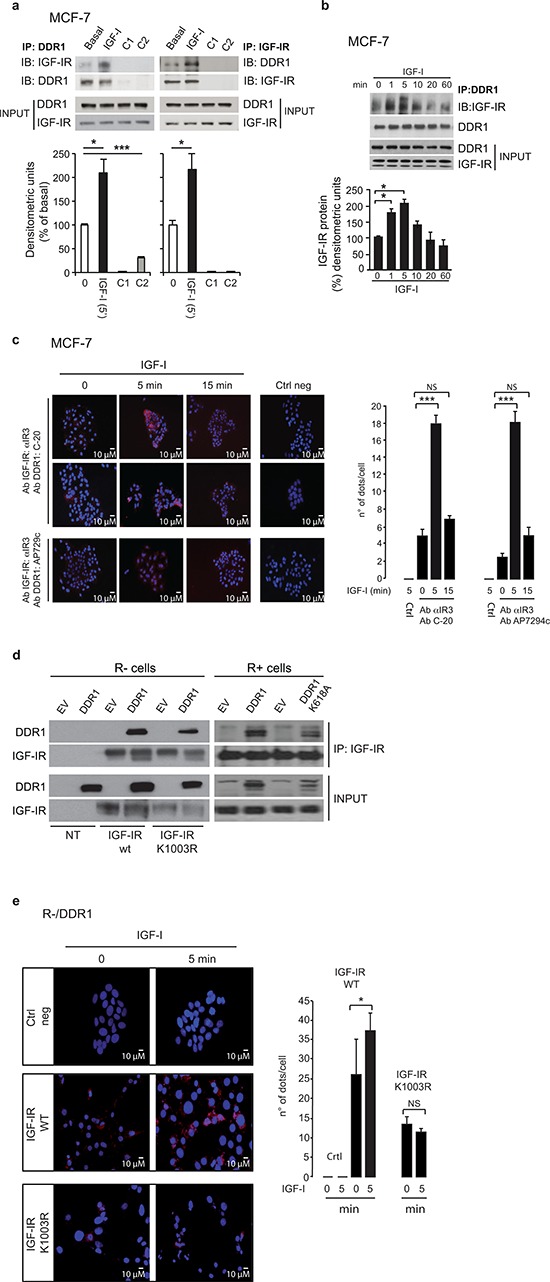
DDR1 associates with the IGF-IR **(a)** *DDR1 and IGF-IR coimmunoprecipitate*. MCF-7 cells were serum starved for 24 h and stimulated with 10 nM IGF-I for 5 min. Cells were then solubilized and lysates were immunoprecipitated with an anti-DDR1 (left panel) or an anti-IGF-IR (αIR3) antibody (right panel), as indicated, and analyzed by immunoblot. Negative controls, including the use of beads only (C1) or of an unrelated primary antibody (C2) (polyclonal anti-HA Y-11, Santa Cruz), are also shown. Total lysates (input) were evaluated as control. Filters were probed with anti-DDR1 and anti-IGF-IR antibodies, as indicated. A representative blot of four independent experiments is shown. Graphs represent the mean ± SEM of four independent experiments, where DDR1 phosphoprotein was normalized for total DDR1 protein immunoprecipitated. ***p < 0.001 (basal *vs*. IGF-I), Student's *t*-test. **(b)** *In vitro DDR1 and IGF-IR co-immunoprecipitation assay*. MCF-7 cells were serum starved for 24 h and then stimulated with IGF-I (10 nM) for the indicated times. Untreated cells are indicated as zero. Samples were then processed as described in Materials and Methods. Filters were probed with anti-DDR1 and anti-IGF-IR antibodies, as indicated. Total lysates (input) were evaluated as control. A representative blot of three independent experiments is shown. Graph represents the mean ± SEM of three independent experiments, where DDR1 phosphoprotein was normalized for total DDR1 protein immunoprecipitated. **(c)** *In situ proximity ligation assay (in situ PLA) confirms DDR1 and IGF-IR association*. *In situ* PLA performed in MCF-7 cells shows that endogenous DDR1 constitutively associates with the IGF-IR. This association significantly increases at 5 min after 10 nM IGF-I stimulation and almost returns at basal levels at 15 min. Two antibody combinations (anti-IGF-IR monoclonal Ab IR3 plus anti-DDR1 polyclonal Ab C-20 and anti-IGF-IR monoclonal Ab αIR3 plus anti-DDR1 polyclonal Ab) gave very similar results. No significant signal was observed with the omission of primary antibody (Ctrl neg). Proteins association is shown as speckled red signals. The histograms (left panel) represent the mean number of dots per high magnification field (150 cells in at least 10 different fields were counted for each conditions). Error bars indicate SEM. Data shown in histograms are from two independent experiments for each antibody combination. ***p < 0.001 (IGF-I *vs*. basal). **(d)** *DDR1 and IGF-IR kinase-inactive co-immunoprecipitate (left panel)*. R^−^ mouse fibroblasts cells expressing either an empty vector (EV) or the human wild-type DDR1 (DDR1/wt) cDNAs were transiently transfected with plasmids coding for either the wild-type IGF-IR (IGF-IR/wt) or the IGF-IR/K1003R mutant. *IGF-IR and DDR1 kinase-inactive coimmunoprecipitate (right panel)*. R^+^ mouse fibroblasts expressing IGF-IR wt, were transiently transfected with plasmids coding for either an empty vector (EV) or the DDR1/K618A mutant cDNAs. 24 h after transfection, cells were lysed and immunoprecipitated with an anti-IGF-IR (αIR3) antibody and analyzed by immunoblot. Lysates of transfected cells were also loaded (input) and immunoblotted with anti-DDR1 and anti-IGF-IR antibodies. A representative of three independent experiments is shown. **(e)** *The association of DDR1 with IGF-IR kinase-inactive is not increased by IGF-I stimulation*. *In situ* PLA performed in R-/DDR1 cells showed that DDR1 association with IGF-IR wild type (WT) increases after 5 min of IGF-I stimulation, while the association between DDR1 and kinase-inactive variant IGF-IR/K1003R does not. No significant signal was observed with the omission of primary antibody. Proteins association is shown as speckled red signals. The histograms (right panel) represent the mean number of dots per high magnification field (150 cells in at least 10 different fields were counted for each conditions). Error bars indicate SEM. Data shown in histograms are from two independent experiments for each condition. NS, *p* > 0.05; *0.01 < *p* < 0.05 (IGF-I *vs*. basal).

In order to evaluate whether the IGF-IR associates with DDR1, MCF-7 cells were subjected to immunoprecipitation in the absence or presence of IGF-I stimulation. As shown in Figure 2a and Figure S1a–1c, IGF-IR and DDR1 associated constitutively, but the association significantly increased after IGF-I stimulation. Data were confirmed by time course studies (Figure 2b), which showed that the association between the IGF-IR and DDR1 readily increased at 1–5 min after IGF-I stimulation.

We further confirmed a direct interaction between DDR1 and IGF-IR by using a proximity ligation assay (*in situ* PLA), which allows quantification and localization of protein-to-protein interactions with single molecule resolution in cells. PLA confirmed that the two molecules interact in intact MCF-7 cells and that this interaction increased after IGF-I stimulation (Figure 2c). No appreciable signal was detected when the specific antibodies were omitted, confirming the specificity of constitutive and IGF-I—stimulated DDR1—IGF-I interaction. In agreement with immunoprecipitation studies, IGF-IR–DDR1 association significantly increased after 5 min IGF-I exposure, and declined after 15 min (Figure 2c).

As shown in transiently transfected R^−^ fibroblasts (Figure 2d, left panel), the constitutive association between IGF-IR and DDR1 was confirmed after expressing a kinase-inactive IGF-IR/K1003R mutant and DDR1 (Figure 2d, left panel). The interaction was also detectable between the IGF-IR and the kinase-inactive DDR1/K618A mutant, which is not phosphorylated upon collagen stimulation [29], as shown in transfected R^+^ cells (Figure 2d, right panel). PLA studies using both IGF-IR wild type and IGF-IR/K1003R mutant indicated that a functional IGF-IR is required to fully sustain IGF-I-enhanced DDR1–IGF-IR interaction (Figure 2e).

Collectively, these results indicate that IGF-IR associates with DDR1 constitutively. However, this association is rapidly enhanced by IGF-I stimulation.

### IGF-I induces DDR1 phosphorylation, and a functional IGF-IR plays an important role in collegen-dependent DDR1 tyrosine-phosphorylation

**Figure 3:**
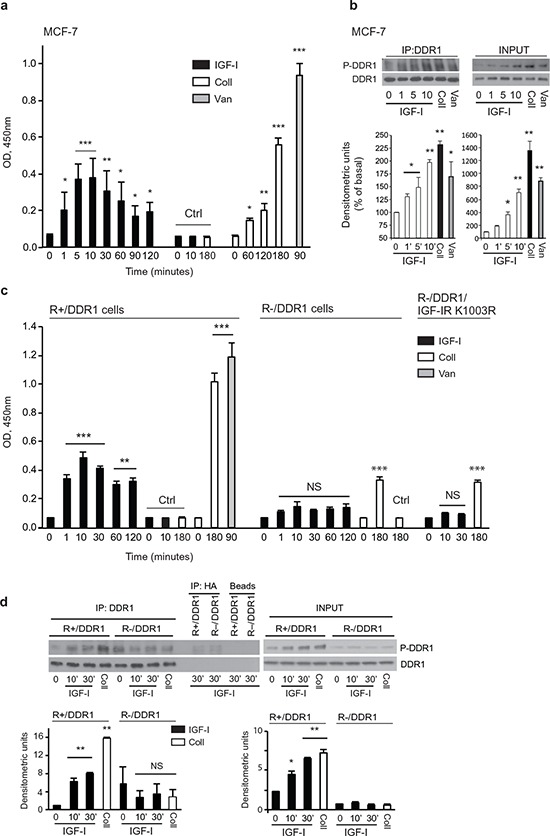
IGF-I induces collagen-independent DDR1 phosphorylation **(a)** and **(b)** *IGF-I induces DDR1 phosphorylation in MCF-7 cells*. (A) MCF-7 cells were serum starved for 24 h and stimulated for the indicated time points with 10 nM of IGF-I. Cell lysates were then used to measure DDR1 phosphorylation with a specific phospho-ELISA assay. Cells exposed to collagen IV 10 μg/ml (Coll) and to Na3VO4 1 mM (Van) were employed as positive controls for DDR1 phosphorylation. Negative controls (Ctrl) were provided by the omission of the primary antibody, either in unstimulated cells (0) or in cells exposed to IGF-I (IGF) or collagen IV (Coll). Graph represents the mean±SD of three independent experiments. (B) Cell lysates obtained as in (A) were used for western blot analysis. Cells exposed to collagen IV 10 μg/ml for 180 min (Coll) and to Na3VO4 1 mM (Van) for 90 min were employed as positive controls for DDR1 phosphorylation. Cell lysates were immunoprecipitated with anti-DDR1 antibody (C20) and then blotted with a specific phospho-DDR1(Tyr792) antibody (left panel). Western blot of whole lysates (*input*) is shown in the right panel. Graphs represent the mean±SEM of densitometric analysis of two independent experiments where P-DDR1 signal was normalized againsttotal DDR1. **(c)** and **(d)** *IGF-I-induced DDR1 phosphorylation requires the IGF-IR*. (c) R^−^ and R^+^ cells were transiently transfected with plasmid encoding DDR1/wt. Cells were serum starved for 24 h and stimulated with IGF-I (10 nM), collagen IV 10 μg/ml (Coll) or to Na3VO4 1 mM (Van) for the indicated times. Cell lysates were then used to measure DDR1 phosphorylation with a specific phospho-ELISA assay. Negative controls (Ctrl) were provided by the omission of the primary antibody, either in unstimulated cells (0) or in cells exposed to IGF-I (IGF) or collagen IV (Coll). Graph represents the mean±SD of three independent experiments. (d) Cell lysates from R^−^/DDR1 and R^+^/DDR1 cells were prepared as in (c), immunoprecipitated with anti-DDR1 antibody (C20) and then blotted with a specific phospho-DDR1(Tyr792) antibody (left panel). Western blot of whole lysates (*input*) is shown in the right panel. Figure shows a representative of two experiments. Graphs represent the mean±SEM of densitometric analysis of two independent experiments after normalization of DDR1 phosphoprotein against total DDR1. (a–d) Statistical significance was calculated using one-way ANOVA followed by Bonferroni test. NS: not significant, *p* > 0.05; *0.05 < *p* > 0.01. **0.001 < *p* < 0.01; ***p < 0.001; (treated cells *vs*. basal).

DDR1 binds to and is activated by various forms of collagen [30, 17, 22] in an integrin-independent fashion [29]. Because DDR1 was present in anti-pY immunoprecipitates from IGF-II stimulated cells [13] and interacted with the IGF-IR (Figure 2) we evaluated whether IGF-I stimulation may affect DDR1 phosphorylation. As shown by ELISA assay (Figure 3a), in MCF-7 cells, DDR1 phosphorylation was barely detectable in unstimulated cells but was significantly induced by IGF-I stimulation peaking at 5–30 min and slowly declining thereafter (Figure 3a). Stimulation with collagen IV (10 μg/ml) and orthovandate (1 mM) was used as positive control. Data were confirmed by western blotting analysis (Figure 3b).

Similar studies were conducted in DDR1-transfected mouse fibroblasts. In R^+^ cells harboring the IGF-IR, DDR1 phosphorylation was induced by IGF-I, with a maximum at 10–30 min, and by collagen IV, as expected (Figure 3c and 3d). On the contrary, in R^−^ cells lacking the IGF-IR, as well as in R^−^ cells transfected with the IGF-IR/K1003R mutant, IGF-I promoted a small but not significant DDR1 phosphorylation (Figure 3c and 3d), which is likely due to IGF-I binding to insulin receptors (IR) expressed in R^−^ cells. Intriguingly, DDR1 phosphorylation in response to collagen IV was also severely impaired in R^−^ and in R^−^/IGF-IR/K1003R cells, although remaining still significant when assessed with the sensitive ELISA assay (Figure 3c). Again, we cannot exclude that DDR1 interaction with IRs expressed in R^−^ cells may play a role in regulating collagen-dependent DDR1 activation in the absence of a functional IGF-IR (Figure 3c and 3d).

These observations are novel and unexpected as they indicate that IGF-I not only induces rapid DDR1 phosphorylation in a collagen-independent fashion, but also that a functional IGF-IR plays a critical role in modulating collagen-dependent DDR1 phosphorylation.

### DDR1 expression levels affect IGF-I mediated biological effects in cancer cells

**Figure 4:**
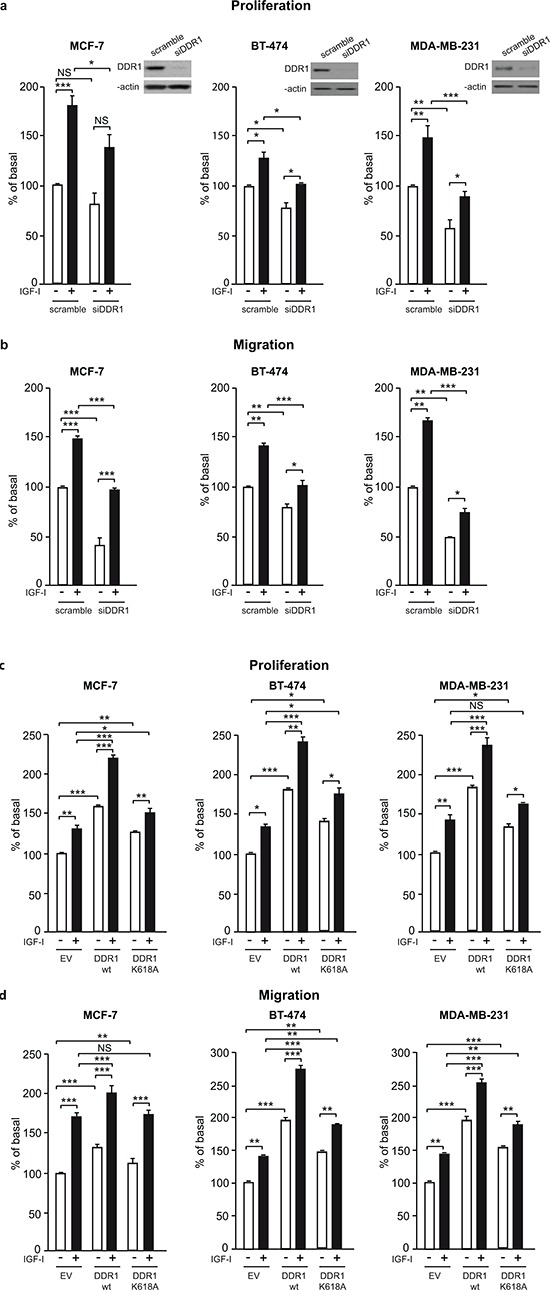
DDR1 expression affects IGF-I mediated biological effects in human cancer cells **(a)** *Cell proliferation after DDR1 silencing.* MCF-7, BT-474 and MDA-MB-231 breast cancer cells were transiently transfected with either a siRNA to DDR1 or scramble siRNAs. After 24 h, cells were grown in medium containing 2.5% of CS-FCS for 24 h and then incubated with or without 10 nM of IGF-I for further 48 h. Cell viability was evaluated by MTT assay. Values are expressed as percentages of untreated scramble oligo-transfected cells (basal) and represent the mean±SEM of three independent experiments in triplicate. NS, *p* > 0.05; *0.01 < *p* < 0.05; **0.001 < *p* < 0.01; ***p < 0.001; (untreated *vs*. IGF-I treated cells in scramble and siDDR1 conditions; untreated scramble *vs*. untreated siDDR1 cells; IGF-I treated scramble *vs*. IGF-I stimulated siDDR1 cells respectively). DDR1 silencing was confirmed for each cells lines by western blot analysis as shown on the right of each histogram. **(b)** *Migration after DDR1 silencing.* MCF-7, BT-474 and MDA-MB-231 breast cancer cells were transiently transfected as in (a) After 24 h, cells were grown in medium containing 0.1% of BSA for additional 24 h. Cells were then removed from plates with 0.01% trypsin and seeded on polycarbonate filters coated with 25 μg/mL fibronectin. Cells were allowed to migrate for 6 h (MCF-7 and MDA-MB-231) or 8 h (BT-474 cells) in response to 10 nM of IGF-I added to the lower chamber. Values are mean±SEM of three independent experiments done in duplicate and are expressed as percent of untreated scramble cells (basal). *0.01 < *p* < 0.05; **0.001 < *p* < 0.01; ***p < 0.001; (untreated *vs*. IGF-I treated cells in scramble and siDDR1 conditions; untreated scramble *vs*. untreated siDDR1 cells; scramble + IGF-I *vs*. siDDR1 + IGF-I). **(c)** *Cell proliferation in DDR1-overexpressing cells.* MCF-7, BT-474 and MDA-MB-231 breast cancer cells were transiently transfected with the wild type or kinase-inactive DDR1 mutant (DDR1/wt or DDR1/K618A) or the corresponding empty vector (EV). After 24 h, cells were grown in medium containing 2.5% of CS-FCS for 24 h and then incubated with or without 10 nM of IGF-I for further 48 h. Cell viability was assessed as in (A) Values are mean±SEM from three independent experiments in duplicate and are expressed as percent of untreated (EV) transfected cells (basal). *0.01 < *p* < 0.05; **0.001 < *p* < 0.01; ***p < 0.001; (untreated *vs*. IGF-I treated cells in EV, DDR1/wt and DDR1/K618A conditions; untreated EV transfected *vs*. untreated DDR1/wt or DDR1/K618A transfected cells; IGF-I treated EV transfected *vs*. IGF-I stimulated DDR1/wt or DDR1/K618A transfected cells). **(d)** *Migration after DDR1 overexpression.* MCF-7, BT-474 and MDA-MB-231 breast cancer cells were transiently transfected as in (c) Cell migration in response to 10 nM of IGF-I was evaluated as in (b) Values are mean±SEM of three independent experiments in duplicate and are expressed as percent of untreated (EV) transfected cells (basal). NS, *p* > 0.05; *0.01 < *p* < 0.05; **0.001 < *p* < 0.01; ***p < 0.001; (untreated *vs*. IGF-I treated cells in EV, DDR1/wt and DDR1/K618A conditions; untreated EV transfected *vs*. untreated DDR1/wt or DDR1/K618A transfected cells; IGF-I treated EV transfected vs. IGF-I stimulated DDR1/wt or DDR1/K618A transfected cells). (a–d) Statistical significance was calculated using one-way ANOVA followed by Bonferroni test.

Activation of the IGF-IR regulates a vast array of biological responses including cell proliferation, migration and protection from apoptosis. We therefore assessed whether DDR1 may modulate IGF-IR-induced biological responses in breast cancer cells. In all three cell lines tested, DDR1 depletion by siRNA approaches resulted in the inhibition of both basal and IGF-I-stimulated proliferation (Figure 4a). DDR1 silencing also strongly affected basal and IGF-I-stimulated cell migration through fibronectin (Figure 4b). It is important to mention that fibronectin is not a DDR1 ligand, indicating that the inhibition of cell migration induced by DDR1 depletion is independent of DDR1 function as a collagen receptor. Similar results were obtained using collagen IV-coated filters (not shown).

Next, we asked whether DDR1 overexpression could also influence IGF-IR-mediated biological responses. Thus, we transiently transfected MCF-7, BT-474 and MDA-MB-231 cells with constructs expressing either wild type DDR1 or the DDR1/K618A mutant, and then evaluated proliferation and migration in response to IGF-I. In all cell lines DDR1 expression significantly increased proliferation (Figure 4c) and migration (Figure 4d) in both untreated and IGF-I-stimulated cells. Expression of the kinase defective DDR1 mutant (DDR1/K618A) was less effective than the expression of wild type DDR1 (DDR1/wt), indicating that these biological effects are partially dependent on DDR1 kinase activity (Figure 4c, 4d). As an additional approach, we used DDR1-IN-1 dihydrochloride, a recently described specific DDR1 tyrosine kinase inhibitor [31]. We confirmed that DDR1-IN-1 inhibits DDR1 phosphorylation in response to collagen IV in a dose dependent manner (Figure S2a). In MCF-7 cells DDR1-IN-1 partially inhibited both basal and IGF-I stimulated proliferation (Figure S2b), confirming data obtained with the DDR1/K618A mutant.

### DDR1 modulates IGF-IR mediated biological effects in non-transformed cells

In order to ascertain whether DDR1 may modulate IGF-IR mediated biological responses also in non-transformed cells, we used R^−^ fibroblasts, which lack *IGF-IR* and express very low levels of endogenous DDR1. These cells are unresponsive to IGF-I and unable to form colonies in soft-agar [32]. However, overexpression of the human IGF-IR restores the ability of these cells (R^+^ cells) to respond to IGF-I, and induces a ligand-dependent transformed phenotype [33].

**Figure 5:**
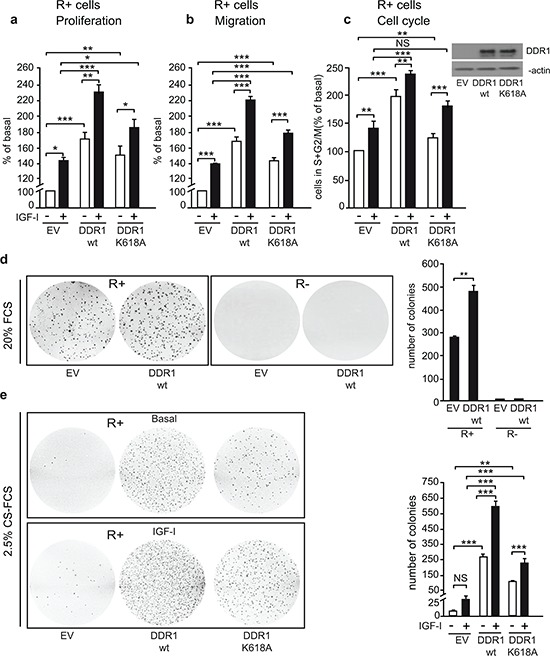
DDR1 expression regulates IGF-I biological effects in non-transformed cells **(a)** *Cell proliferation after DDR1 overexpression*. R^+^ mouse fibroblasts transfected with plasmids encoding either wild type DDR1 (DDR1/wt) or the DDR1/K618A mutant or the corresponding empty vector (EV), were plated in 96-well plates. 24 h after plating, cells were grown in medium containing 2.5% CS-FCS for 24 h and then incubated with or without IGF-I (10 nM) for additional 48 h. Cell viability was evaluated by MTT assay. Values are expressed as percentage of untreated (EV) transfected cells (basal) and represent the mean±SEM of three independent experiments performed in triplicate. *0.01 < *p* < 0.05; **0.001 < *p* < 0.01; ***p < 0.001; (untreated *vs*. IGF-I treated cells in EV, DDR1/wt and DDR1/K618A conditions; untreated EV transfected cells *vs*. untreated DDR1/wt or DDR1/K618A transfected cells; IGF-I treated EV transfected cells *vs*. IGF-I stimulated DDR1/wt or DDR1/K618A transfected cells). **(b)** *Migration after DDR1 overexpression*. R^+^ mouse fibroblasts transfected with plasmids encoding either the DDR1/wt or the DDR1/K618A mutant or the corresponding empty vector (EV) were grown in medium containing 0.1% of BSA for 24 h. Cells were then removed from plates with 0.01% trypsin and seeded on polycarbonate filters coated on the upper side with 25 μg/mL fibronectin. Cells were allowed to invade for 6 h in response to 10 nM IGF-I added to the lower chamber. Values are mean±SEM of three independent experiments done in duplicate and are expressed as percent of untreated (EV) transfected cells (basal). *0.01 < *p* < 0.05; **0.001 < *p* < 0.01; ***p < 0.001; (untreated *vs*. IGF-I treated cells in EV, DDR1/wt and DDR1/K618A conditions; untreated EV transfected cells *vs*. untreated DDR1/wt or DDR1/K618A transfected cells; IGF-I treated EV transfected cells *vs*. IGF-I stimulated DDR1/wt or DDR1/K618A transfected cells). **(c)** *Cell cycle progression after DDR1 overexpression*. R^+^ mouse fibroblasts transfected with plasmids encoding either the DDR1/wt or the DDR1/K618A mutant or the corresponding empty vector (EV) were grown in medium containing 0.1% of BSA for 24 h. Cells were then incubated with or without IGF-I (10 nM) for additional 48 h and analyzed for their cell-cycle profiles. Cell populations positive for propidium iodine staining were evaluated by FACS analysis, and G0/G1 and G2/M phases were scored. The graph shows the percentage of cells in S and G2/M phases. Values are expressed as percent of basal (untreated EV transfected cells) and are the mean±SEM of three independent experiments. NS, *p* > 0.05; **0.001 < *p* < 0.01; ***p < 0.001; (untreated *vs*. IGF-I treated cells in EV, DDR1/wt and DDR1/K618A conditions; untreated EV transfected cells *vs*. untreated DDR1/wt or DDR1/K618A transfected cells; IGF-I treated EV transfected cells *vs*. IGF-I stimulated DDR1/wt or DDR1/K618A transfected cells). **(d)** *Colony formation after DDR1 overexpression.* R^−^ and R^+^ mouse fibroblasts stably transfected with plasmids encoding either the DDR1/wt or the corresponding empty vector (EV), were seeded in soft-agar, as described in Materials and Methods. Cells were plated in triplicate and grown in complete medium containing 20% FCS for 3 weeks. Colonies were stained with methyl thiazolyl tetrazolium (MTT) and then photographed. The *histogram* represents the mean number of colonies shown in (d) Error bars indicate SEM (*n* = 3 dishes). Data shown in (d) are from two independent experiments. **0.001 < *p* < 0.01; (EV *vs*. DDR1/wt). **(e)** *Colony formation after DDR1 overexpression in response to IGF-I.* R^+^ mouse fibroblasts stably transfected with plasmids encoding either the DDR1/wt or the DDR1/K618A mutant or the corresponding empty vector (EV), were seeded in soft-agar. Cells were plated in triplicate and cultured in serum free medium containing 2.5% CS-FCS for 3 weeks. Colonies were stained with methyl thiazolyl tetrazolium (MTT) and then photographed. The *histogram* represents the mean number of colonies shown in (E) Error bars indicate SEM (*n* = 3 dishes). NS, *p* > 0.05; **0.001 < *p* < 0.01; ***p < 0.001; (untreated *vs*. IGF-I treated cells in EV, DDR1/wt and DDR1/K618A conditions; untreated EV transfected cells *vs*. untreated DDR1/wt or DDR1/K618A transfected cells; IGF-I treated EV transfected cells *vs*. IGF-I stimulated DDR1/wt or DDR1/K618A transfected cells). (a–e) Statistical significance was calculated using one-way ANOVA followed by Bonferroni test.

As expected, in R^+^ cells, IGF-I stimulated cell growth, migration, and cell cycle progression (Figure 5a–5c). All these IGF-I-induced responses were significantly enhanced by transfection with wild type DDR1 (Figure 5a–5c), while transfection with the DDR1/K618A mutant had a reduced effect as compared to wild type DDR1 (Figure 5a–5c). DDR1 overexpression also enhanced basal growth and migration of R^+^ cells, but not of R^−^ cells (Figure S3a–S3b), suggesting that these effects are likely due to some constitutive activation of overexpressed IGF-IR in R^+^ cells.

When seeded in 20% FCS-containing soft-agar, R^+^ cells formed a discrete number of colonies, which significantly increased in size and number upon DDR1 overexpression (Figure 5d). In contrast, DDR1 overexpression in R^−^ fibroblasts had no effect (Figure 5d) on colony formation.

To further confirm that this DDR1-dependent effect required IGF-IR activation, we seeded R^+^ or R^+^/DDR1 cells in 2.5% CS-FCS-containing soft-agar in the presence or absence of IGF-I. Serum-starved R^+^ cells formed very few small colonies, which were moderately enhanced in size and number after IGF-I stimulation (Figure 5e). In contrast, R^+^/DDR1 cells were more clonogenic, and colony size and number were greatly enhanced by IGF-I stimulation (Figure 5e). Importantly, transfection of a DDR1/K618A mutant was less effective than wild type DDR1 (Figure 5e) in enhancing colonies size and number.

These experiments clearly demonstrate that DDR1 enhances IGF-I-induced cell biological responses in a collagen-independent manner. Notably, in R^+^ cells DDR1 strongly enhanced the acquisition of an IGF-I–dependent transformed phenotype. The presence of a functional IGF-IR was essential for DDR1 action.

### DDR1 is rapidly co-internalized with the IGF-IR after IGF-I stimulation and affects IGF-IR trafficking

Having established that DDR1 is an important modulator of IGF-IR function, we sought to gain further insight into the possible mechanisms involved. After ligand-induced phosphorylation, the IGF-IR is subjected to rapid internalization, sorting into early endosomes followed by degradation or recycle to the cell membrane [34, 35]. In MCF-7 and other cancer cells, a small pool of IGF-IR translocates into the nucleus [36]. Regulation of IGF-IR trafficking and intracellular localization affects receptor function and IGFs-mediated biological responses [35, 36].

**Figure 6:**
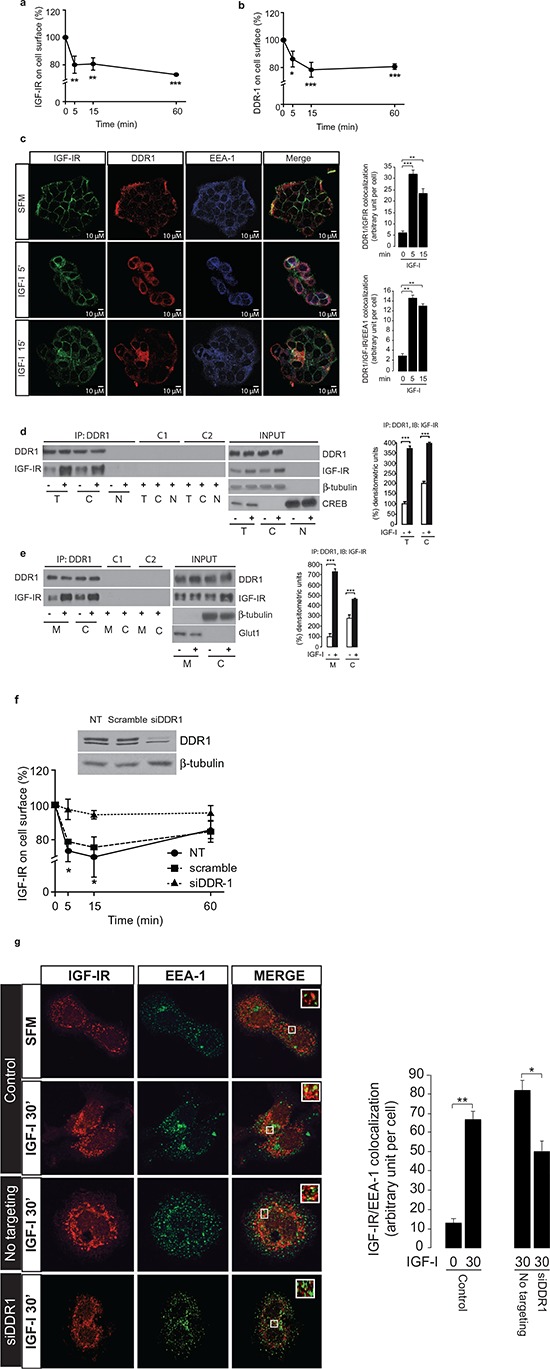
DDR1 co-internalizes with IGF-IR and affects IGF-IR trafficking **(a–b)** *ELISA analysis of IGF-IR and DDR1 internalization*. MCF-7 cells were stimulated with IGF-I (10 nM) and the level of cell surface IGF-IR and DDR1 were determined by ELISA assay, as described in Methods, at different time points of stimulation. **(c)** *IGF-IR and DDR1 co-localize to endosomes.* MCF-7 cells were plated onto cover slips and serum-starved for 24 h. Cells were then stimulated with IGF-I (10 nM) for the indicated times. The triple staining indicating co-localization of the IGF-IR with DDR1 and EEA-1 was assessed by confocal microscopy. Colocalization index was calculated by ImageJ software. **(d)** and **(e)** IGF-I stimulation increases IGF-IR-DDR1 association at the cytoplasm (d) and membrane (e) level. MCF-7 cells were serum starved for 24 h and stimulated with 10 nM IGF-I for 5 min. Cells were then solubilized and total lysates **(t)**, cytoplasmic (c) and membrane **(m)** fractions were immunoprecipitated with anti-DDR1 (C-20) (upper panels). Negative controls, including the use of an unrelated primary antibody (anti-HA, Y-11) or beads only are also shown. An aliquot of each fraction (input) was evaluated as control. Filters were probed with anti-DDR1 or anti-IGF-IR antibodies, as indicated. Anti β-tubulin, CREB and GLUT1 were used to respectively confirm cytoplasm, nuclear and membrane purification, respectively. A representative blot of four independent experiments is shown. Graphs represent the mean ± SEM of four independent experiments, where co-immunoprecipitated IGF-IR was normalized for the immunoprecipitated total DDR1 protein. ***p < 0.001 (basal *vs*. IGF-I), Student's *t*-test. **(f)** *IGF-IR internalization is affected by DDR1 silencing*. MCF-7 cells were transiently transfected with siRNA to DDR1 or scramble siRNAs. After 48 h, cells were stimulated with IGF-I (10 nM), and the level of cell surface IGF-IR was determined by ELISA. Untransfected cells are indicated as NT. DDR1 silencing was assessed by immunoblot analysis shown on the right of the ELISA graph. Data are the average ± SEM of three independent experiments. Statistical significance was determined using two-way ANOVA and Bonferroni post-test. *0.01 < *p* < 0.05; **0.001 < *p* < 0.01; ***p < 0.001. **(g)** *IGF-IR localization to endosomes is affected by DDR1 silencing*. MCF-7 cells were plated onto cover slips and transiently transfected with siRNA to DDR1 or scramble siRNAs. After 48 h, cells were stimulated with IGF-I (10 nM) for 30 min. Colocalization of the IGF-IR with EEA-1 was assessed by confocal microscopy. Insets represent enlarged views (3 ×) of boxed region. One hundred cells from at least 10 independent fields were examined. Images were collected on a Leica TCS-SP2 confocal microscope as described in Methods. Images were merged using Photoshop CS4. Pictures are representative of three independent experiments.

Thus, we first assessed whether DDR1 may play any role in regulating IGF-IR endocytosis, and analyzed the internalization rate of IGF-IR and DDR1 by measuring MCF-7 cell surface receptors after IGF-I stimulation [34, 35]. As previously demonstrated [34, 35], IGF-I exposure induced a clear reduction of IGF-IR cell surface levels at 5–60 min (Figure 6a). Significantly, IGF-I also induced a significant internalization of DDR1 with a similar kinetics (Figure 6b). We then used immunofluorescence (IFL) confocal microscopy to follow proteins localization. In untreated MCF-7 cells, DDR1 and IGF-IR were mainly detectable at the plasma membrane where they partially co-localized (Figure 6c). After IGF-I exposure, both the IGF-IR and DDR1 were rapidly internalized and, at 5 min, mostly localized in early endosomes as demonstrated by IGF-IR colocalization with the early endosomal marker EEA-1. Cell fractionation studies confirmed that the IGF-IR and DDR1 constitutively associate in cell membrane and cytosolic fractions and, after stimulation with IGF-I, association increased in both fractions (Figure 6d and 6e). The IGF-IR and DDR1 were not detected in nuclear fractions (Figure 6d), likely due to the small pools of receptors possibly translocating to the nucleus in these experimental conditions.

Importantly, DDR1 depletion by siRNA significantly reduced IGF-IR internalization (Figure 6f) and determined a clear reduction of IGF-IR detectable in early endosomes (Figure 6g) confirming the critical role of DDR1 in regulating IGF-IR internalization and sorting into early endosomes. Taken together, these results indicate that DDR1 regulates IGF-IR trafficking and intracellular localization, which may impact on IGF-IR-mediated biological responses.

### DDR1 increases IGF-IR protein expression at post-translational level

**Figure 7:**
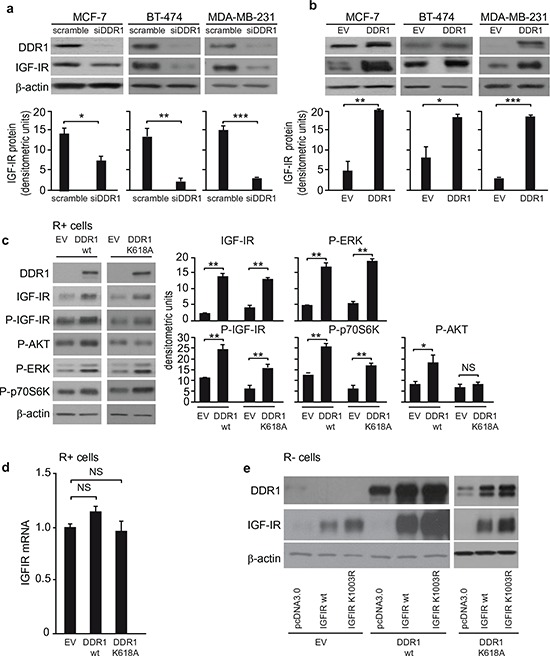
DDR1 level affects IGF-IR protein expression **(a)** *IGF-IR protein expression after siDDR1 silencing*. Breast cancer cells were transiently transfected with siRNA to DDR1 or scramble siRNAs. After 72 h, cells were lysed and analyzed by SDS-PAGE and immunoblotted with the indicated primary antibodies. β-actin was used as control for protein loading. Blot is representative of three independent experiments. The histograms represent the mean±SEM of densitometric analysis of three independent experiments. Statistical significance was determined using Student's *t*-test. NS, *p* > 0.05; *0.01 < *p* < 0.05; **0.001 < *p* < 0.01; ***p < 0.001; (scramble *vs*. siDDR1 conditions). **(b)** *IGF-IR protein expression after DDR1 overexpression*. Breast cancer cells were transiently transfected with plasmids encoding either the human wild-type DDR1 (DDR1/wt), or the corresponding empty vector (EV). After 72 h, cells lysed and analyzed by SDS-PAGE and immunoblotted with the indicated primary antibodies. β-actin was used to control for protein loading. The top panels show a representative experiment. The histograms represent the mean±SEM of densitometric analysis of three independent experiments after normalization against β-actin. Statistical significance was determined using Student's *t*-test. NS, *p* > 0.05; *0.01 < *p* < 0.05; **0.001 < *p* < 0.01; ***p < 0.001; (EV *vs*. DDR1 transfected cells). **(c)** *IGF-IR protein expression and downstream signaling after DDR1 overexpression in R^+^*cells**. R^+^ fibroblasts stably transfected with plasmids encoding either DDR1/wt or the DDR1/K618A mutant or the corresponding empty vector (EV), were lysed, analyzed by SDS-PAGE and immunoblotted with the indicated primary antibodies. β-actin was used to control for protein loading. The top panel shows a representative experiment. The histograms represent the mean±SEM of densitometric analysis of three independent experiments after normalization against β-actin. Statistical significance was determined using Student's *t*-test. NS, *p* > 0.05; *0.01 < *p* < 0.05; **0.001 < *p* < 0.01; ***p < 0.001; (EV *vs*. DDR1/wt or DDR1/K618A). **(d)** *IGF-IR mRNA levels after DDR1 silencing in R^+^*cells**. IGF-IR mRNA levels were evaluated in the same cells shown in (C) Normalization was done using β-actin as housekeeping control gene. Data are presented as the mean ± SEM of three independent experiments. Statistical significance was determined using one-way ANOVA. NS, *p* > 0.05; (EV *vs*. DDR1/wt *vs*. DDR1/K618A). **(e)** *IGF-IR protein expression after DDR1 overexpression in R^−^*cells**. R^−^ mouse fibroblasts stably expressing either the human DDR1/wt or the DDR1/K618A mutant or the corresponding empty vector (EV) were transiently transfected with plasmids encoding for either the IGF-IR/wt or the IGF-IR/K1003R mutant. 24 h after transfection, cells were lysed and analyzed by immunoblot.β-actin was used for control of protein loading. The panel shows a representative of three of independent experiments.

As DDR1 affects IGF-IR trafficking and downstream signaling, we asked whether it might also modulate IGF-IR protein expression. DDR1 silencing was accompanied by a reduction of IGF-IR protein levels in all three breast cancer cell lines tested (Figure 7a) while DDR1 overexpression was associated with enhanced IGF-IR levels, which were especially marked in MCF-7 and MDA-MB-231 cells (Figure 7b). In MCF-7 cells IGF-IR mRNA levels remained unchanged after DDR1 silencing or overexpression (not shown), indicating that DDR1 affects IGF-IR expression at post-translational levels.

We confirmed these results in transfected R^+^ cells, where IGF-IR protein expression markedly increased after transfection with wild type DDR1 or DDR1/K618A mutant, as compared to mock-transfected control cells (Figure 7c). Accordingly, IGF-IR autophosphorylation and downstream signaling were also significantly enhanced by wild type DDR1 expression, but less affected by the DDR1/K618A mutant (Figure 7c). As in MCF-7 cells, DDR1 expression did not significantly affect IGF-IR mRNA in spite of increasing IGF-IR protein (Figure 7d). In addition, DDR1 expression also increased the level of the kinase defective IGF-IR/K1003R mutant (Figure 7e). Furthermore, the DDR1/K618A mutant was still able to enhance the expression of both IGF-IR/wt and IGF-IR/K1003R (Figure 7e). Conversely, DDR1 protein levels increased in IGF-IR transfected cells (Figure 7e).

Taken together, these data indicate that DDR1 modulates IGF-IR signaling and biological responses by regulating IGF-IR internalization and intracellular sorting. In addition, DDR1 modulates IGF-IR protein expression levels by a post-translational regulatory mechanism.

### DDR1 regulates IGF-IR downstream signaling

**Figure 8:**
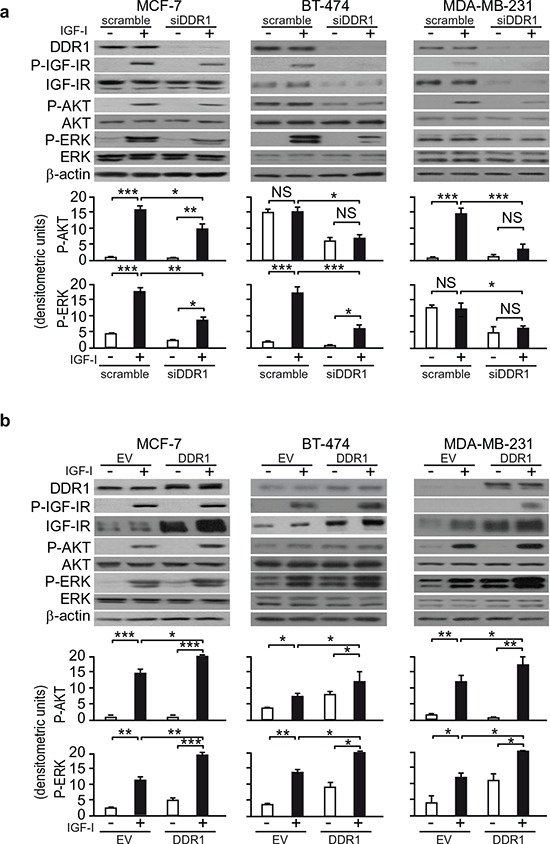
DDR1 expression level affects IGF-I downstream signaling in human breast cancer cells **(a)** *IGF-I signaling after DDR1 silencing*. MCF-7, BT-474 and MDA-MB-231 breast cancer cells were transiently transfected with siRNA to DDR1 or scramble siRNAs. After 48 h, cells were grown in medium containing 2.5% of CS-FCS for 24 h and then stimulated with or without 10 nM of IGF-I for 5 min. Cells were then lysed and analyzed by SDS-PAGE and immunoblotted with the indicated primary antibodies. β-actin was used as control for protein loading. The top panels show a representative of three experiments. The histograms represent the mean±SEM of densitometric analysis of three independent experiments after normalization of each phosphoprotein against β-actin. Statistical significance was determined using one-way ANOVA. NS, *p* > 0.05; *0.01 < *p* < 0.05; **0.001 < *p* < 0.01; ***p < 0.001; (untreated *vs*. IGF-I treated cells in scramble and siDDR1 conditions; untreated scramble *vs*. untreated siDDR1 cells; scramble + IGF-I *vs*. siDDR1 + IGF-I). **(b)** *IGF-I signaling after DDR1 overexpression.* MCF-7, BT-474 and MDA-MB-231 breast cancer cells were transiently transfected with plasmids encoding either human wild-type DDR1 (DDR1/wt) or the corresponding empty vector (EV). After 48 h, cells were grown in medium containing 2.5% of CS-FCS for 24 h and then stimulated with or without 10 nM of IGF-I for 5 min. The activation of downstream signaling was assessed as in (a) Blot is representative of three independent experiments. The histograms represent the mean ±SEM of densitometric analysis of three independent experiments after normalization of each phosphoproteins against β-actin. Statistical significance was determined using one-way ANOVA. NS, *p* > 0.05; *0.01 < *p* < 0.05; **0.001 < *p* < 0.01; ***p < 0.001; (untreated *vs*. IGF-I treated, after cell transfection with EV, DDR1/wt and DDR1/K618A; untreated, EV *vs*. DDR1/wt or DDR1/K618A transfected cells; IGF-I stimulated, EV vs. DDR1/wt or DDR1/K618A).

Because DDR1 affected IGF-IR protein expression levels, as well as IGF-IR internalization and trafficking which are critical steps in fine-tuning the intensity of receptor signaling, we then evaluated whether DDR1 could affect IGF-IR activation and downstream signaling. In ER positive MCF-7 and BT-474 cells as well as in triple negative MDA-MB-231, DDR1 silencing significantly reduced IGF-IR autophosphorylation and both AKT and ERK1/2 phosphorylation in response to IGF-I (Figure 8a). Conversely, DDR1 overexpression enhanced IGF-IR autophosphorylation and both AKT and ERK1/2 activation in response to IGF-I in all three cell lines (Figure 8b). In DDR1-transfected MDA-MB-231 and R^+^ cells, time course studies after stimulation with IGF-I confirmed the increase in phosphorylation of IGF-IR and downstream signaling (Figure S4A and S4B). These data clearly indicate that DDR1, by acting at multiple levels, significantly modulates the two main signaling cascades downstream of the IGF-IR in breast cancer cells and transfected fibroblasts.

## DISCUSSION

Our present results show that DDR1 associates with IGF-IR at the cell membrane, and that DDR1 is rapidly tyrosine-phosphorylated and internalized with the IGF-IR upon IGF-I stimulation. Within minutes from IGF-I stimulation, IGF-IR and DDR1 co-localize in early endosomes and at the perinuclear region. These data are novel, as in fact DDR1, a collagen receptor, has not been previously implicated in cross-talk with the IGF-IR. Moreover, DDR1 is known to be tyrosine-phosphorylated only after binding to different forms of collagen (collagen I, IV and VI) and with slow kinetics, which requires hours of collagen stimulation. However, it has been recently reported that, following collagen stimulation, DDR1 is rapidly internalized and incorporated into early endosomes, similarly to other receptor tyrosine kinases [21]. It seems, therefore, that DDR1 internalization and phosphorylation are two events that, in part, are temporally and spatially separated [21]. Here we show for the first time that, after IGF-I stimulation, DDR1 is rapidly co-internalized with the IGF-IR and is required for IGF-IR internalization and localization into early endosomes. Therefore, DDR1 appears to have a novel scaffolding role for IGF-IR. Noteworthy, IGF-IR localization at the level of early endosomes has recently emerged as an important mechanisms of signal transduction [37]. We also showed that DDR1 is able to enhance IGF-IR protein expression by a post-translational regulatory mechanism. Further studies are required to address whether DDR1 affects IGF-IR stability by regulating IGF-IR sorting for degradation or by other mechanisms.

DDR1 positively modulates several IGF-I-dependent biological actions, namely cell proliferation, migration and colony formation. These peculiar effects of DDR1 are not associated with its collagen binding activity, as they occur in the absence of collagen. They appear to be only partially dependent on DDR1 kinase activity, as they are somewhat shared by the kinase-inactive DDR1/K618A mutant, which was in fact able to enhance IGF-IR protein expression and signaling, although at a lower extent than DDR1 wild type. These data support a possible scaffolding role for DDR1 in regulating IGF-IR signals, independent of collagen stimulation of DDR1 kinase activity. However, IGF-I dependent stimulation of DDR1 kinase activity and IGF-IR–DDR1 association may further enhance IGF-IR downstream signaling and biological responses.

Interestingly, DDR1 affected not only maximal rates of proliferation, migration and colony formation after IGF-I stimulation, but also basal rates. However, the presence of a functional IGF-IR was absolutely necessary, as demonstrated by the absence of DDR1 effect in R^−^ cells lacking the IGF-IR. It is well known that cancer and IGF-IR overexpressing cells show some basal IGF-IR tyrosine kinase activity, which may explain the effect of DDR1 in regulating to some extent responses in the absence of IGF-I stimulation. Notably, we also report for the first time that DDR1 phosphorylation in response to collagen is severely impaired in the absence of a functional IGF-IR. More studies are required to establish whether the residual DDR1 phosphorylation in response to collagen might be mediated through IGF-I binding to insulin receptors. DDR1 expression also increased in the presence of IGF-IR, a phenomenon that we are currently investigating in our laboratory. Although our study is the first one revealing a functional interaction between DDR1 and IGF-IR, *DDR1* knock out mice were previously found to be smaller in size than control littermates [24], indicating a role of DDR1 in growth and development. Moreover, there is evidence that DDR1 is overexpressed in a variety of malignancies, which have also a deregulated IGF axis [25]. In particular, DDR1 is among the more prominent molecules expressed by sarcomas characterized by constitutive IGF-II overexpression [38]. In light of our results, it can be hypothesized that concomitantly high levels of DDR1 and IGF-IR may play an important role in cancer progression. IGF-IR activation may likely contribute to DDR1 protumorigenic activity, which includes upregulation of matrix metalloproteases 1, 2, and 9, activation of NF-kB and other relevant signaling pathways [16, 22]. We found that DDR1a and DDR1b are almost equally expressed in breast cancer cells. These isoforms are both able to dimerize and to be activated by collagen binding [27]. Whether they elicit different biological effects is poorly understood, although it has been reported that in neutrophils only DDR1a but not DDR1b stimulates migration [26]. Therefore, there is the possibility that the relative abundance of DDR1 isoforms in the various tissues may differently affect the biology of the IGF-IR—DDR1 cross-talk.

In summary, this study demonstrates that, in addition to its well-recognized role of collagen receptor, DDR1 acts as a novel scaffolding molecule for the IGF-IR, and modulates several IGF-IR—mediated biological responses in both transformed and non-transformed cells by affecting IGF-IR protein expression, trafficking, and signaling. In turn, IGF-IR plays a major role in DDR1 phosphorylation by collagen. DDR1 emerges, therefore, as an important modulator of IGF-IR functions, while IGF-IR appears to regulate interactions with the microenvironment through DDR1. In addition, these data suggest that this DDR1-IGF-IR functional cross-talk may play a role in cancer progression thereby establishing DDR1 as an important novel therapeutic target in cancers associated with a dysregulated IGF-IR pathway.

## MATERIALS AND METHODS

### Materials

IGF-I was purchased from Prepotech (Rocky Hill, NJ); Bovine Serum Albumin (BSA), fibronectin, collagen Type I and IV from Sigma Aldrich (St. Louis, MO); Metafectene PRO from Biontex Laboratories GmbH (Germany); lipofectamine 2000, lipofectamine RNAiMax, Opti-MEM, fetal calf serum (FCS), Geneticin (G-418) from Life Technologies, Inc. Laboratories (Paisley, UK). Constructs encoding either an empty vector (pCMV6-Entry vector) or the human wild type DDR1 isoform a (DDR1wt) cDNAs were from OriGene (Rockville, MD, USA). The DDR1 mutant K618A (DDR1/KD) in the pCMV6-Entry vector was generated with the QuikChange II XL Site-Directed Mutagenesis Kit (Agilent Technologies).

Plasmids encoding for either wild-type IGF-IR (IGF-IR/wt) or the kinase dead IGF-IR/K1003R mutant (IGF-IR/KD) cloned into pcDNA3.0 vector were kindly provided by Dr. R. O'Connor (University College, Cork, Ireland). The specific silencer Select Pre-designed siRNA for DDR1 (Human DDR1 siGENOME SMARTpool Cat M-003111–04) and the negative control siRNA were from Thermo Fisher Scientific Dharmacon (NYSE:TMO). DDR1 tyrosine kinase inhibitor DDR1-IN-1 dihydrochloride was obtained by Tocris Bioscience (Bristol, UK).

### Cell cultures

The human cancer cell lines MCF-7, T47D, ZR-75, MDA-MB-157, MDA-MB-231, BT-474, HepG2 and rat myoblasts L6 were purchased from the American Cell Type Culture Collection and cultured according to the manufacturer's instructions. R^−^ cells, mouse embryo fibroblasts with targeted disruption of the *IGF-IR* gene were kindly provided by Dr. R. Baserga (Kimmel Institute, Philadelphia). The R^+^ cells were derived from R^−^ cells by stable transfection with the human IGF-IR cDNA. Both cell lines were generated and maintained as previously described [39]. R^−^/EV, R^−^/DDR1wt, R^−^/DDR1/K618A as well as R^+^/EV, R^+^/DDR1wt and R^+^/DDR1/K618A clones were generated by transfecting R^−^ and R^+^ cells with pCMV6-Entry vector or the human wild type DDR1 isoform a (DDR1wt) or the mutant DDR1/K618A, respectively. After transfection, cells were selected in G-418 containing medium and subcloned.

### Real-time PCR

Total RNA (2 μg) was reversely transcribed using the ThermoScript RT (Invitrogen) and oligo(dT) primers. Synthesized cDNA was combined in a PCR reaction using primers for the gene of interest (see Table 1). Relative quantitative quantification of target genes was done by comparing ΔCt, as previously described [40].

**Table 1:** Primers used for quantitative PCR

h-DDR1 total	Fw 5′ gcgtctgtctgcgggtagag 3′ Rv 5′ acggcctcagataaatacattgtct 3′
h-DDR1 isoform a	Fw 5′ ccccaatggctctgccta 3′ Rv 5′ aacaatgtcagcctcggcata 3′
h-DDR1 isoform b	Fw 5′ ggccaaacccaccaacac 3′ Rv 5′ aacaatgtcagcctcggcata 3′
m-DDR1 total	Fw 5′ tcaccatcaaaatcgccgac 3′ Rv 5′ CTGGCTGTTGTGAACTTCCC 3′
m-GAPDH	Fw 5′ TGACGTGCCGCCTGGAGAAA 3′ Rv 5′ AGTGTAGCCCAAGATGCCCTTCAG 3′
h-β-actin	Fw 5′ GACAGGATGCAGAAGGAGATCACT 3′ Rv 5′ TGATCCACATCTGCTGGAACCT 3′
IGF-IR	Fw 5′ TGGTGGAGAACGACCATATCC 3′ Rv 5′ CGATTAACTGAGAAGAGGAGTTCGA 3′

Fw, Forward; Rv, Reverse

### Immunoprecipitation

Cells were lysed and processed as previously described [40]. The following antibodies were used for immunoprecipitation: anti-DDR1 (C-20, sc-532, Santa Cruz Biotechnology) and anti-IGF-IR clone αIR3 (Merck Chemicals, Nottingham, UK). The following antibodies were used for western blotting: anti-IGF-IR (sc-713) and anti-DDR1 (C-20, sc-532) (Santa Cruz Biotechnology).

### *In situ* proximity ligation assay (PLA)

MCF-7 cells were plated onto coverslips, serum-starved for 24 h, and then stimulated or not with IGF-I for 5 min. Slides were fixed with 4% paraformaldehyde, permeabilized with 0.1% Triton X-100, and stained according to the manufacturer (Olink Bioscience, Uppsala, Sweden). After blocking, slides were incubated with anti-DDR1 and anti-IGF-IR antibodies, overnight at 4°C. Experiments were carried out with two antibody combinations: a) anti-IGF-IR monoclonal Ab αIR3 (recognizing the cysteine-rich region of the extracellular α-chain) plus anti-DDR1 polyclonal Ab C-20 (epitope mapping at the C-terminus of DDR1 of human origin); b) anti-IGF-IR monoclonal Ab αIR3 plus anti-DDR1 polyclonal Ab (recognizing epitope between 299–330 amino acids from the central region of human DDR1).

After washing, the anti-mouse and anti-rabbit PLA probes were added for 1 h, and the ligation mixture added for 30 min followed by the amplification-polymerase mixture. Finally, slides were washed and mounted in 4′,6-diamidino-2-phenylindole (DAPI) containing medium. Cells were analyzed and photographed on a Leica TCSSP2 confocal microscope with a X63 Apo PLA oil immersion objective (NA 1.4) and 60 μm aperture using the LEICA Scan TCS-SP2 software (Leica Microsystems, Wetzlar, Germany). Pictures are representative of 150 cells in at least 10 independent fields from four independent experiments. Images were analyzed with NIH ImageJ.

### Western blot analysis

Subconfluent cells were lysed and subjected to western blot analysis, as previously described [28]. To evaluate IGF-I-dependent activation of downstream signaling after DDR1 silencing or overexpression, cells were serum-starved for 24 h, and then stimulated with IGF-I (10 nM) for 5 min. The following antibodies were used: anti-DDR1, anti-IGF-IR and anti-β-tubulin (Santa Cruz Biotechnology); anti-P-IR/P-IGF-IR (Y1150/Y1151), anti-IGF-IR, anti-P-Akt (S473), anti-AKT, anti-P-ERK1/2 (T202/Y204), anti-ERK1/2, anti-P-DDR1 Tyr792 (Cell Signaling Technology); anti-P-DDR1 Tyr513 (Origene); anti-β-actin (Sigma Aldrich); anti-phosphotyrosine antibody (4G10) (Upstate, Biotechnology).

### Gene silencing by small interfering RNA, and gene overexpression

For small interfering RNA (siRNA) experiments, cells were transfected with a mixture containing Opti-Mem, Lipofectamine RNAiMax and either 10 nM scramble siRNA or siRNA specific for DDR1. For overexpression experiments, cells were transfected with a mixture containing the DNA of interest, Opti-Mem, lipofectamine 2000 or MetafectenePro. After 24 h, cells were serum starved for 24 h, and stimulated with IGF-I (10 nM) for the indicated times.

### Cell viability assay

Cell viability was measured by the methyl thiazolyl tetrazolium (MTT) test (Amersham Biosciences). Cells were transiently transfected with the indicated plasmids or siRNAs. 24 h after transfection, cells were grown in medium containing 2.5% CS-FCS for 24 h followed by IGF-I exposure (10 nM) for further 48 h. The cells were then incubated with medium containing 0.5 mg/ml MTT, and processed as previously described [40].

### Migration assay

The ability of cells to invade fibronectin or collagen IV was measured in Boyden's chambers. Cells were transiently transfected with the indicated plasmids or siRNA. 24 h after transfection, cells were serum starved for 24 h, removed from plates with 0.01% trypsin and placed on polycarbonate filters (8 μm pore size, Corning Costar), coated with 250 μg/mL collagen IV or 25 μg/mL fibronectin. Cells were allowed to migrate for 6–8 h in response to 10 nM IGF-I [28].

### Cell cycle evaluation

Cells synchronized for 24 h in serum-free medium were exposed to IGF-I (10 nM) for 48 h and subjected to fluorescence-activated cell sorting (FACS) analysis, as previously described [28].

### Soft-agar colony formation assay

Anchorage-independent growth was assessed as previously described [41]. Briefly, cells suspended in medium containing 20% FCS (or 2.5% CS-FCS when stimulated with IGF-I) and 0.3% agar, were plated on top of the bottom layer agar (20% FBS and 0.8% agar). Top agar was then covered with culture medium with or without IGF-I (10 nM). Stimulus was changed twice a week and cells were cultured for 3 weeks. Colonies were visualized with 0.5 mg/mL MTT, photographed and analyzed with NIH ImageJ.

### Internalization assay

Cell surface receptors were assessed by ELISA, as previously described [35, 42]. Briefly, cells were plated in 10% FCS medium, serum starved for 24 h and incubated in the absence or presence of 10 nM of IGF-I for various time points. Cells were fixed in 3.7% formaldehyde containing Tris-buffered saline (TBS), washed with TBS, and blocked with 1% BSA-TBS. Cells were then incubated for 1 h with a monoclonal anti-DDR1 N-terminal (Asp19-Thr416) antibody (R&D Sistem (Clone 290420) MAB2396 or anti-IGF-IR monoclonal antibody αIR3, washed, re-blocked, and incubated for 1 h with goat anti-mouse alkaline phosphatase-conjugated antibody. After cell washing, antibody binding was visualized by adding 0.25 ml of alkaline phosphatase substrate. Plates were read at 405 nm in a microplate reader.

### Confocal microscopy

MCF-7 cells were plated onto coverslips, serum-starved for 24 h, and then stimulated with IGF-I. Coverslips were processed for immunofluorescence and confocal analysis, as previously described [43]. Antibodies used were: anti-IGF-IR monoclonal antibody αIR3 (Millipore), anti-DDR1 polyclonal antibody C-20 (Santa Cruz), anti-EEA-1 monoclonal antibody (R&D System). Secondary antibodies were: Alexa Fluor 488 (green), Alexa Fluor 594 (red) and Alexa Fluor 647 (purple) (Molecular Probes). Coverslips were analyzed and photographed on a Leica TCSSP2 confocal microscope with a X63 Apo PLA oil immersion objective (NA 1.4) and 60 μm aperture using the LEICA Scan TCS-SP2 software. Pictures are representative of at least 10 independent fields from three independent experiments. Fields were selected for the presence of cells with the following criteria: well defined limits, clear identification of nucleus and absence of intersection with neighboring cells. An average of 100 cells was examined for each condition. Data are representative of ~80% of the total number of cells examined. Co-localization index was calculated using NIH ImageJ software.

### Cell fractionation studies

#### Subcellular Fractionation

MCF-7 cells were washed twice with PBS and then mechanically detached with PBS. One third of the cells were centrifuged and solubilized in RIPA buffer for the total fraction. The remaining two thirds were centrifuged at 1200 rpm for 10 min at 4°C. Pellets for fractionation were resuspended in hypotonic buffer (10 mM Tris, pH 8.0, 10 mM KCl, 2 mM phenylmethylsulfonyl fluoride plus protease inhibitor mixture) to allow cell swelling for 2 min at 4°C. Then, Nonidet P-40 was added to a final concentration of 0.4%. Samples were centrifuged at 2,000 rpm for 5 min at 4°C and the supernatants collected as the cytoplasmic fractions. Pellets containing cell nuclei were washed once with hypotonic buffer and then extracted with high salt lysis buffer (50 mM Tris pH 8.0, 5 mM EDTA, pH 8.0, 1% Nonidet P-40, 400 mM NaCl, 2 mM phenylmethylsulfonyl fluoride plus protease inhibitor mixture) and sonicated. Equal amounts of proteins were loaded onto SDS acrylamide gel, transferred onto nitrocellose membranes, and blotted with anti-DDR1 (C-20), anti IGF-IR (Cell Signaling Technology), anti-β-tubulin, and anti-CREB 48H2 (Cell Signaling Technology) antibodies.

#### Membrane isolation

Membrane proteins were extracted from adherent cells by ProteoExtract Transmembrane Protein Extraction Kit (Novagen, Millipore). Briefly, cells were washed twice with PBS at 4°C, mechanically detached using a cell scraper, and centrifuged at 1000 g for 5 min at 4°C. Pellets were resuspended in extraction buffer 1 with protease inhibitor cocktail, incubated for 10 min at 4°C with gentle agitation and then centrifuged at 1000 g for 5 min at 4°C. Supernatants were collected as the ‘cytosolic’ protein fractions. Pellets were resuspended in extraction buffer 2B with protease inhibitor cocktail, incubated for 45° at room temperature with gentle agitation, and centrifuged at 16,000 g for 15 min at 4°C. Supernatants were collected as the ‘membrane’ protein fractions. Protein concentration of the cytosolic and membrane protein fractions were measured with the BCA assay.

### ELISA for DDR1 phosphorylation

DDR1 phosphorylation was evaluated by PathScan^®^ Phospho-DDR1 (panTyr) Sandwich ELISA (Cell Signaling), following the manufacturer's instructions. Briefly, cells were plated in 10% FCS medium, serum starved for 24 h, and then incubated in the absence or presence of either IGF-I (10 nM) or Type IV collagen. Na3VO4 was used as positive control. Nonspecific signal was provided by the omission of the primary antibody. Cells lysates were incubated o/n at 4°C in the appropriate wells. After washing, the P-Tyr antibody was added to wells, and the phospho-DDR1 visualized with HRP-linked secondary antibody and TMB substrate. Plates were read at 450 nm in a microplate reader.

### Densitometric and statistical analysis

Densitometry results were obtained with NIH ImageJ. Differences between means were evaluated by one- or two-way ANOVA followed by post-hoc analysis of significance (Bonferroni test) for the comparison between more than two groups, whereas the Student's *t*-test for unpaired samples was used for comparisons between two groups. The level of significance was set at *p* < 0.05. Statistical analysis was performed with GraphPad Prism5 (GraphPad Software, San Diego, CA). Data were expressed as mean ± SEM.

## SUPPLEMENTARY FIGURES


