# Supplementary material for: Novel cross-talk between IGF-IR and DDR1 regulates IGF-IR trafficking, signaling and biological responses
Source: Oncotarget. 2015 Mar 28;6(18):16084–105. doi: 10.18632/oncotarget.3177 (PMC4599258; doi:10.18632/oncotarget.3177)
Supplement: Supplementary file 1 [file oncotarget-06-16084-s001.pdf]

SUPPLEMENTARY FIGURES

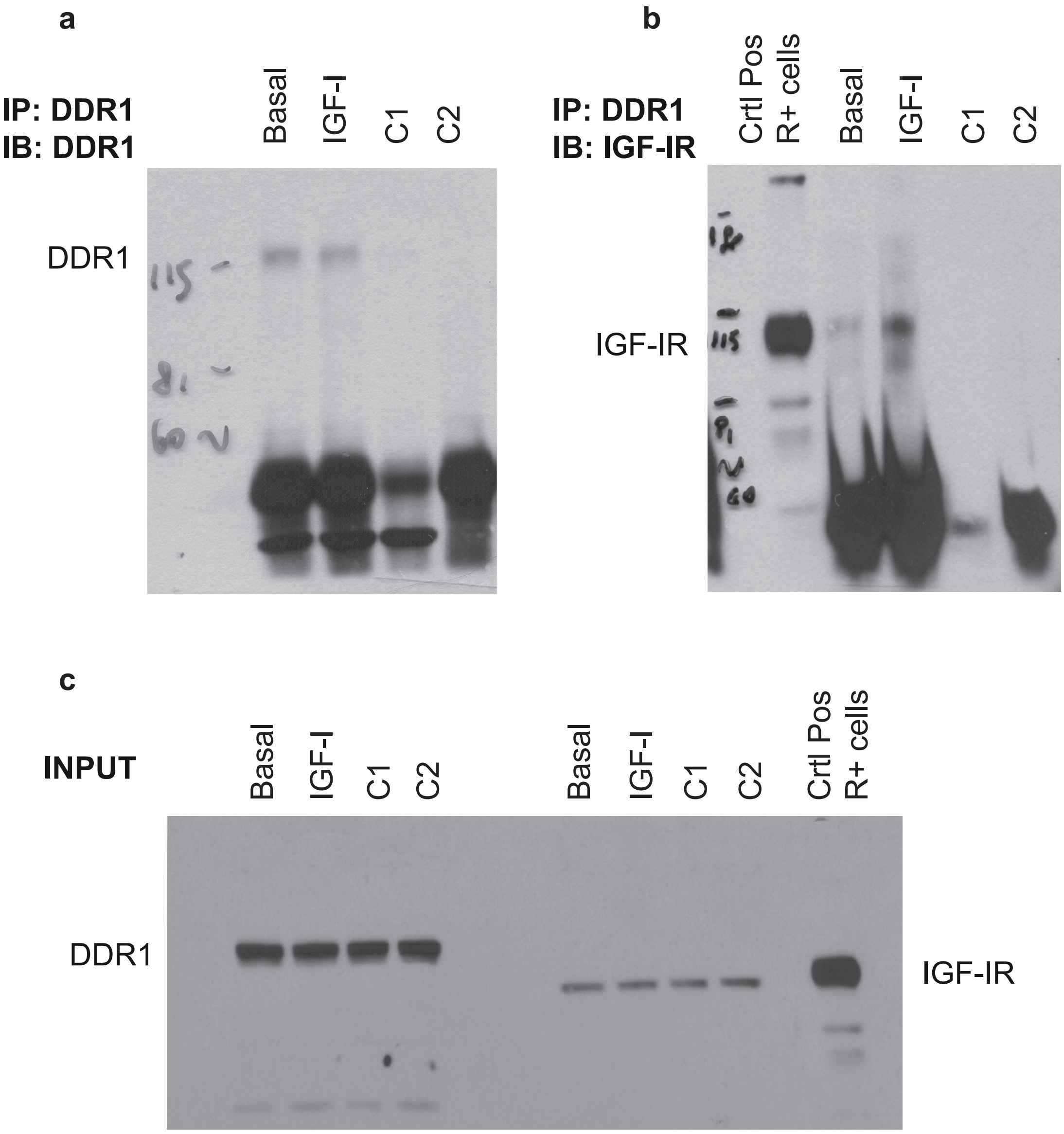

Supplementary Figure S1: DDR1 and IGF-IR coimmunoprecipitation. MCF-7 cells were serum starved for 24 h and stimulated with 10 nM IGF-I for 5 min. Cells were then solubilized and lysates were immunoprecipitated with an anti-DDR1 and analyzed by immunoblot for DDR1 **(a)** or for IGF-IR **(b)**. Negative controls, including the use of beads only (C1) or of an unrelated primary antibody (C2) are also shown. Total lysates (input) were evaluated as control and immunoblotted for DDR1 and IGF-IR **(c)**. Filters were probed with anti-DDR1 and anti-IGF-IR antibodies, as indicated. A representative entire blot of four independent experiments is shown.

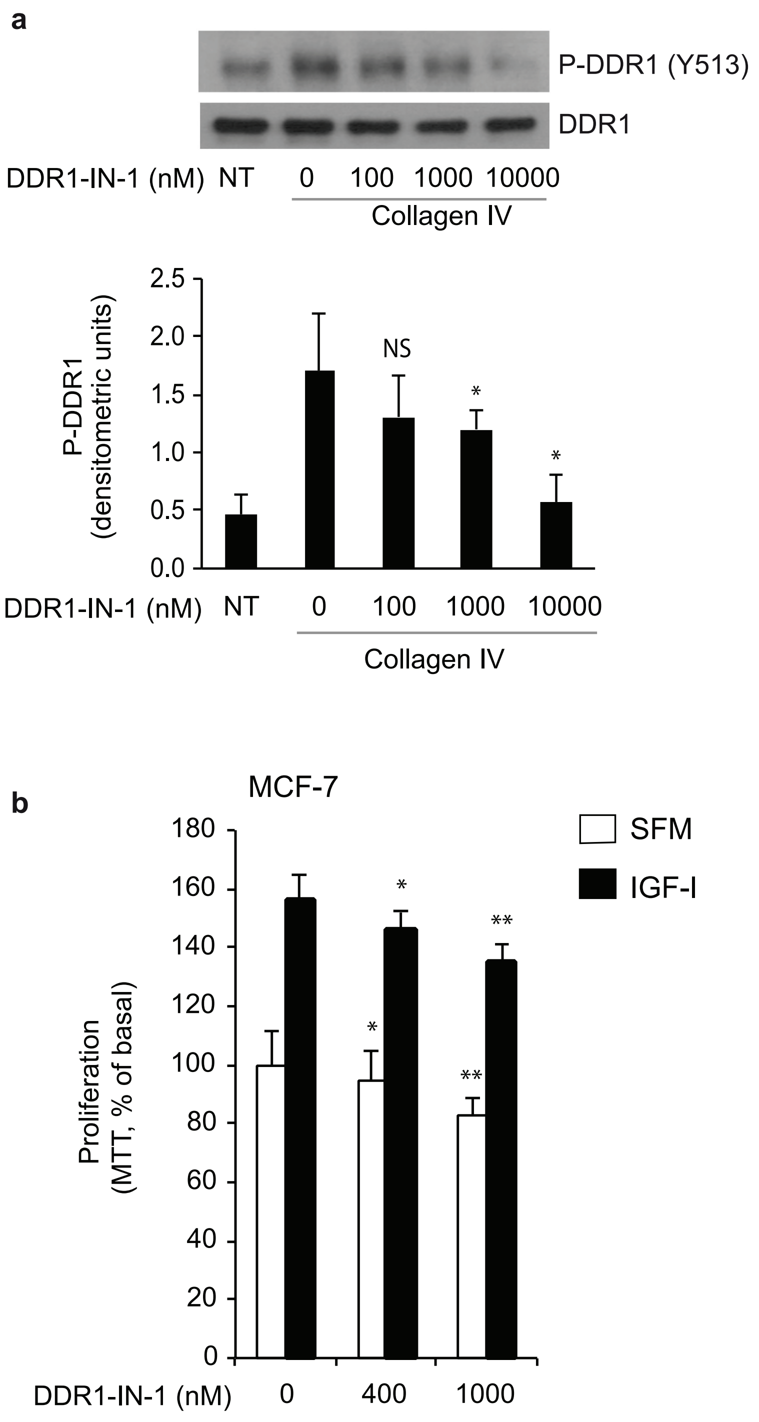

Supplementary Figure S2: Effect of DDR1 tyrosine kinase inhibitor on MCF-7 cell proliferation in response to IGF-I. (a) *DDR1 tyrosine kinase inhibitor DDR1-IN-1 effect on collagen induced DDR1 phosphorylation.* MCF-7 cells were serum starved for 24 h and stimulated with collagen IV 10 µg/ml for 180 min in the absence or in the presence of DDR1-IN-1 at the indicated doses. DDR1 phosphorylation was measured by western blot with the phosphospecific antibody P-DDR1 Tyr513. The graph represents the mean±SEM of densitometric analysis of three independent experiments after normalization of phospho-signals against DDR1. Statistical significance was determined using one-way ANOVA with Bonferroni test. NS, $p > 0.05$; *$0.01 < p < 0.05$. **(b)** *Cell proliferation and IGF-I response in MCF-7 cells exposed to DDR1-IN-1*. MCF-7 cells were grown in medium containing 2.5% CS-FCS for 24 h and then incubated with or without IGF-I (10 nM) for additional 48 h in the absence or the presence of DDR1-IN-1 at the indicated doses. Cell viability was evaluated by MTT assay. Values represent the mean±SEM of six independent experiments performed in quadruplicate. Statistical significance was determined using one-way ANOVA with Bonferroni test. NS, $p > 0.05$; *$0.01 < p < 0.05$; **$0.001 < p < 0.01$ (*DDR1-IN-1* treated *vs*. *DDR1-IN-1* untreated cells in basal conditions and IGF-I treated cells).

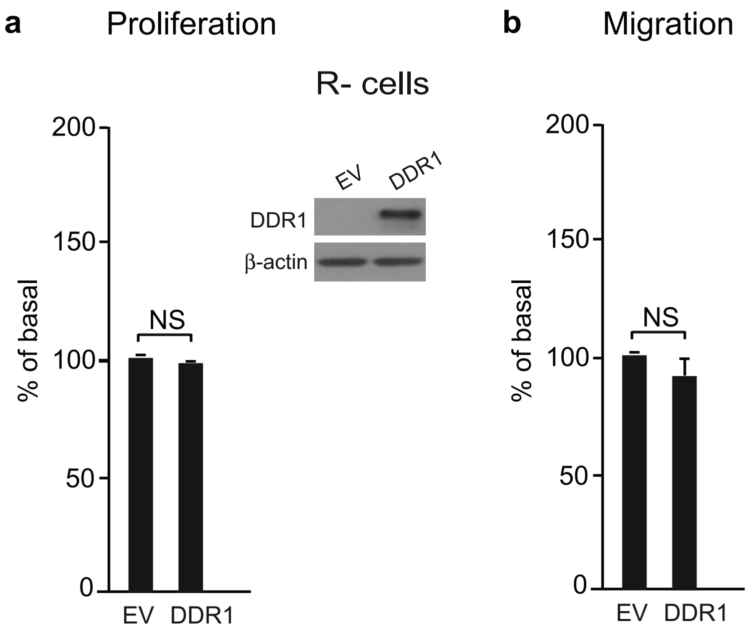

Supplementary Figure S3: IGF-I biological effects in R- cells after DDR1 overexpression. (a) *Cell proliferation*. R⁻ mouse fibroblasts transfected with plasmids encoding either the DDR1/wt or the corresponding empty vector (EV), were plated in 96-well plates, and allowed to grow in complete medium for additional 72 h. Cell viability was evaluated by the MTT assay. Values are expressed as percentage of untreated (EV) transfected cells (basal) and represent the mean±SEM of three independent experiments performed in triplicate. NS, $p > 0.05$ (EV *vs*. DDR1 transfected cells). **(b)** *Migration assay*. R⁻ mouse fibroblasts transfected as in (a) were grown in complete medium for 24 h. Cells were then removed from plates with 0.01% trypsin and seeded on polycarbonate filters coated on the upper side with 25 µg/mL fibronectin. Cells were allowed to migrate for 6 h. Values are means±SEM of three independent experiments done in duplicate and are expressed as percent of untreated (EV) transfected cells (basal). Statistical significance was determined using the Student's *t*-test. NS, $p > 0.05$ (EV *vs*. DDR1 transfected cells). DDR1 overexpression was confirmed, for both (a) and (b) experiments, by western blot analysis as shown beside each histogram.

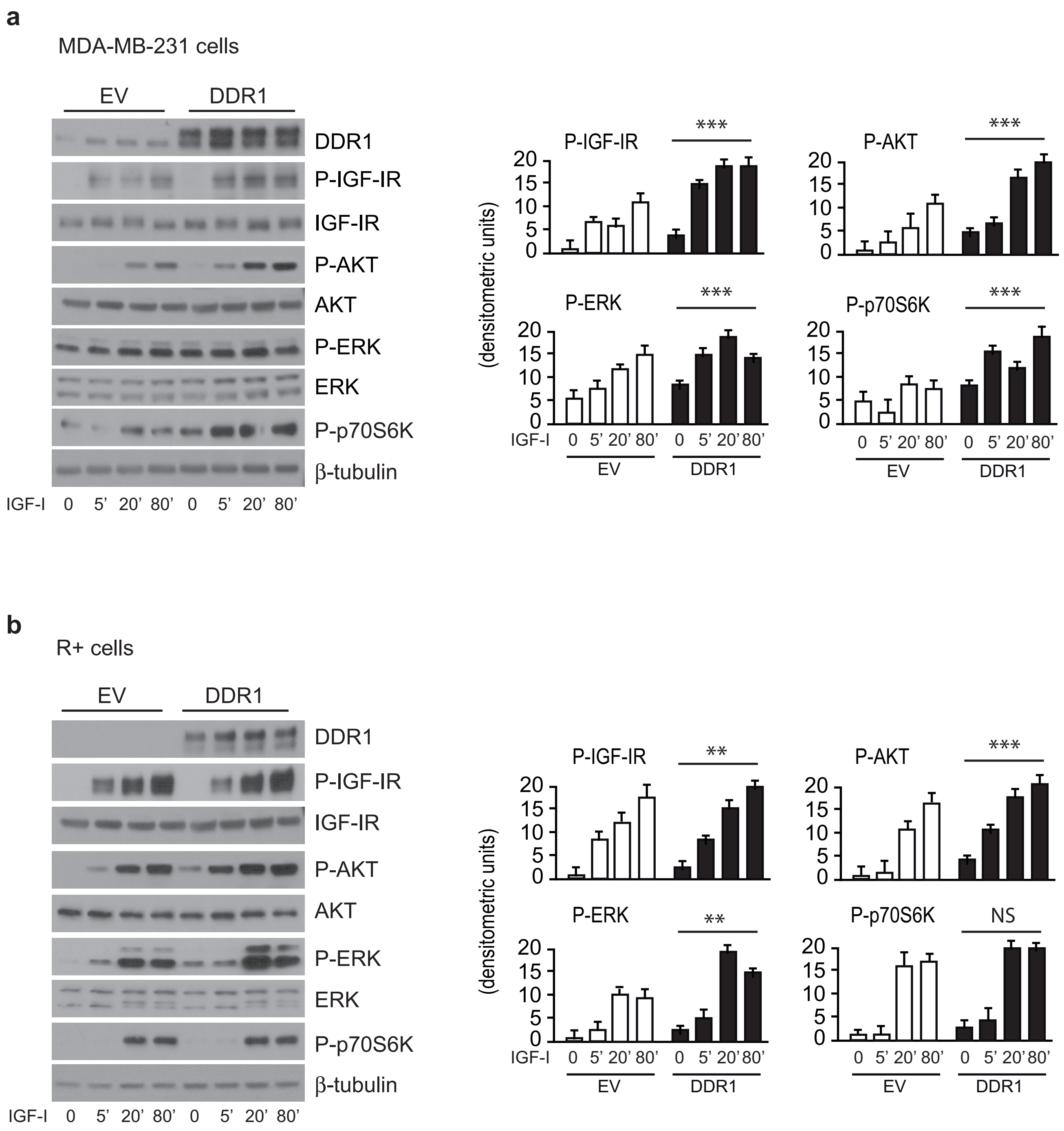

Supplementary Figure S4: DDR1 expression level affects IGF-I downstream signaling in human cancer cells. (a–b) *Kinetics of downstream IGF-I signaling in DDR1 transfected cells.* MDA-MB-231 (a) and R[+] cells (b) were transiently transfected with plasmids encoding either human wild-type DDR1 (DDR1) or the corresponding empty vector (EV). After 48 h, cells were grown in medium containing 2.5% of CS-FCS for 24 h and then stimulated with or without 10 nM of IGF-I for the indicated time points. The activation of downstream signaling was assessed as described in Methods. The blots shown are representative of two independent experiments. The histograms represent the mean±SEM of densitometric analysis of two independent experiments after normalization against each protein. Statistical significance was determined using two-way ANOVA. NS, $p > 0.05$; *$0.01 < p < 0.05$; **$0.001 < p < 0.01$; ***$p < 0.001$; (EV *vs*. DDR1 transfected).
